# Locomotion as a Measure of Well-Being in Captive Chimpanzees (*Pan troglodytes*)

**DOI:** 10.3390/ani13050803

**Published:** 2023-02-23

**Authors:** Sarah Neal Webb, Steven Schapiro

**Affiliations:** 1 Department of Comparative Medicine, Michale E. Keeling Center for Comparative Medicine and Research, The University of Texas MD Anderson Cancer Center, Houston, TX 78602, USA; 2 Department of Experimental Medicine, The University of Copenhagen, Nørregade 10, 1165 Copenhagen, Denmark

**Keywords:** chimpanzee welfare, locomotion, positive welfare

## Abstract

**Simple Summary:**

Captive chimpanzee locomotion, including walking, climbing, brachiating, and hanging, is a species-typical behavior and increased locomotion is generally considered to indicate improved welfare. However, the relationship between locomotion and welfare is nuanced, and there is a limited number of studies that have used locomotion as a welfare indicator. We summarize findings from four previously published studies that showed increased locomotion in captive chimpanzees under social and physical environmental conditions is associated with enhanced welfare, including increases in space per animal and changes in the type of housing, housing in larger and more diverse groups, and during participation in an experimental medication choice paradigm. New data also showed that higher levels of locomotion are related to higher levels of behavioral diversity (an indicator of positive welfare), and lower levels of abnormal behavior and inactivity (behavioral indicators of negative welfare). Given this evidence, we suggest that time spent in locomotion can be used as a sensitive measure of welfare in captive chimpanzees.

**Abstract:**

Locomotion in non-human primates, including walking, climbing, and brachiating among other types of movement (but not pacing), is a species-typical behavior that varies with age, social housing conditions, and environmental factors (e.g., season, food availability, physical housing conditions). Given that captive primates are typically observed to engage in lower levels of locomotor behaviors than their wild counterparts, increases in locomotion are generally considered to be indicative of improved welfare in captivity. However, increases in locomotion do not always occur with improvements in welfare, and sometimes occur under conditions of negative arousal. The use of time spent in locomotion as a welfare indicator in studies of well-being is relatively limited. We conducted focal animal observations on 120 captive chimpanzees across a series of studies and found higher percentages of time spent in locomotion (1) upon transfer to a new enclosure type, (2) in larger groups with wider within-group age ranges, and fewer males, and (3) with participation in an experimental medication choice paradigm. We also found that, among geriatric chimpanzees, those housed in nongeriatric groups exhibited more locomotion than those living in geriatric groups. Lastly, locomotion was significantly negatively correlated with several indicators of poor welfare and significantly positively correlated with behavioral diversity, one indicator of positive welfare. Overall, the increases in time spent in locomotion observed in these studies were part of an overall behavioral pattern indicative of enhanced welfare, suggesting that an increase in time spent in locomotion itself may be an indicator of enhanced welfare. As such, we suggest that levels of locomotion, which are typically assessed in most behavioral experiments, may be used more explicitly as indicators of welfare in chimpanzees.

## 1. Introduction

There are various definitions of locomotion in studies of animal behavior, but all refer to the movement of the animal in relation to the environment [1,2]. In chimpanzees, locomotion refers to movement from one location to another as a result of walking, climbing, jumping, or brachiating. It is a species-typical behavior that varies in frequency and duration among wild chimpanzees according to season and availability of food. Individuals travel between sites within their home range or territory to obtain food, access sleeping sites, and find areas that promote other activities necessary for their survival [3]. Importantly, locomotion in captivity must be distinguished from pacing, which is used as an indicator of negative welfare. Pacing is distinct from locomotion in both form and function, as pacing occurs in a repetitive and stereotypical fashion and occurs with no apparent objective or goal [4,5,6]. We want to make it clear that the behavior we refer to throughout this manuscript is locomotion, and not pacing.

Locomotion accounts for approximately 12–15% of the daily time budget of wild chimpanzees [3,7], whereas locomotion accounts for 5–10% of observation time in studies of captive chimpanzees [8,9,10,11,12,13,14,15,16]. Given that levels of locomotion are lower in captive chimpanzees than in their wild counterparts, and increases in species-typical behaviors in captive populations are typically equated with increases in well-being, increases in locomotion are generally considered to be related to positive welfare [8,9,16,17,18,19,20].

Although locomotion is a behavior of interest in many studies, it is not often explicitly used as a measure of NHP welfare or well-being, at least not to the extent that other behavioral indicators of welfare are used (e.g., abnormal behaviors) [10,11,19,21]. “Gold-standard” indicators of negative well-being [22] include abnormal, stereotypic, and self-directed behavior (and sometimes rough-scratching) [22]. However, there are very few “gold-standard” behavioral indicators of positive welfare in chimpanzees. Social play is often used to indicate positive welfare, but this behavior occurs at low frequencies (approximately 0.5–2% of daily time budget) in adult geriatric, and/or female chimpanzees [9,10,21] (Neal Webb, unpublished data), making it a difficult behavior to use for welfare assessments of an aging chimpanzee population. Furthermore, social play may have a more complicated relationship with positive welfare than has been posited in the past, as adult chimpanzees may use social play to cope with social tension [23]. Other studies have used the level, rate, or presence/absence of species-typical behaviors to make inferences about positive welfare states. For chimpanzees, this includes behaviors such as tool use, nesting, and grooming [24,25]. In a comprehensive survey examining behavioral indices of welfare in facilities housing chimpanzees across the United States, Bloomsmith and colleagues asked about the presence or absence of four species-typical behaviors (i.e., tool use, nesting, grooming, and copulation) and seven abnormal behaviors (including self-directed activity) to provide a snapshot of the state of chimpanzee care and management [24]. Although the authors described the ways that increased activity levels can reflect enhanced welfare, they did not include activity or locomotion in their survey. This illustrates the currently dominant approach to chimpanzee welfare assessment: the use of abnormal behaviors as indicators of negative welfare and species-typical behaviors as indicators of positive welfare. As mentioned above, locomotion likely falls into the category of species-typical behaviors, yet is not commonly used as an indicator of positive welfare [26].

Previous studies have examined locomotion in relation to well-being through changes to the physical environment, such as increased enclosure size or complexity, with associated increased time spent in locomotion [8,9,16,17,18,19,27]. Other studies have included locomotion as a component within a composite behavioral measure, making it difficult to determine the effects of experimental manipulations on locomotion itself. For example, captive chimpanzees showed decreased activity (including locomotion, exploration, and play) and agitation, as well as increased inactive/relaxed behavior, following the playback of different types of music [28]. The authors suggested that this pattern of behavioral change (including decreased activity) meant that the music had a calming effect on the chimpanzees, thereby enhancing their well-being. However, it is unknown whether locomotion increased or decreased, as the authors did not examine the individual behaviors within the composite measure. Still, other studies found increased locomotion that seemed to be consistent with enhanced well-being but did not explicitly make that connection. For example, Jensvold and colleagues reported an increase in certain types of locomotion after chimpanzees were transferred to a larger, more complex enclosure [19]. The authors suggested that the housing improvements that increased locomotion (indicative of increased species-typical behavior) are important elements in the quality of life of captive primates but did not explicitly link locomotion to welfare. Lastly, several studies have examined inactivity as a measure of welfare, finding decreased inactivity as a function of enrichment, transfers to new enclosures, social housing conditions, etc. [9,10,11,17,21,27]. However, decreases in chimpanzee inactivity do not necessarily occur in conjunction with increases in locomotion [9,19]. As such, inactivity levels cannot be used as a perfect proxy for levels of locomotion. Decreased inactivity suggests increased activity, but this increased activity may or may not include increased locomotive behavior.

Further complicating the relationship between locomotion and welfare is the fact that increased locomotion has been found in negatively arousing contexts, or conjunction with negative welfare-related behaviors. For example, chimpanzees that were singly-housed long-term showed higher amounts of time spent in locomotion, aggression toward humans, tension-related behaviors, and scratching compared to socially-housed chimpanzees [8]. The authors suggested that increased stress led to higher levels of locomotion, indicating decreased welfare. Furthermore, increased locomotion under conditions of negative arousal has been found in other NHP species. In rhesus monkeys (*Macaca mulatta*), locomotion increased following maternal separation, a stress-induction paradigm. This increased locomotion coincided with increased cortisol and lower social play, both of which are indicative of decreased well-being [29,30]. Additionally, Erikson and colleagues found that locomotion was highest in rhesus macaques characterized as “high withdrawal”, compared to those classified as low and moderate in withdrawal behavior [29]. Furthermore, this increased locomotion coincided with increases in self-directed behaviors, and long-term separations were associated with decreased locomotion. In marmosets (*Callithrix jacchus*), several studies have shown that locomotion increases following the presentation of a predator in an experimental paradigm [31,32,33,34]. Although these studies were performed in other NHP species, it is likely that similar responses would be seen in chimpanzees.

Given the lack of a clear connection between locomotion and welfare in the existing literature, we aimed to further refine the utility of locomotion as an indicator of positive welfare in captive chimpanzees. Here, we first summarize findings from four previously published studies focused on changes to, or characteristics of, the physical and social environment that included locomotion as a dependent variable. We then present unpublished correlational data between locomotion and welfare-related behaviors.

## 2. Materials and Methods

### 2.1. Subjects

A total of 120 chimpanzees housed at the National Center for Chimpanzee Care (NCCC), The University of Texas MD Anderson Cancer Center (UTMDACC), Michale E. Keeling Center for Comparative Medicine and Research (KCCMR) in Bastrop, Texas were included in this study. The Keeling Center has been continuously accredited by AAALAC-I for over 40 years. At the conclusion of data collection, chimpanzees were housed in 17 separate social groups ranging in size from three to 10 animals per group. There were 73 females and 47 males (M age = 31.39 years, age range = 15–56), 15 of whom were wild-born (unknown rearing history), 27 were nursery-reared, and 78 were mother-reared [15].

Chimpanzees were housed in Primadomes™ (KIVA, Buda, TX, USA) or corrals with indoor/outdoor access. Both Primadomes™ and corrals included various physical environmental enrichment items, including, but not limited to, wooden climbing structures with platforms, telephone poles, culvert sections, tractor tires, various sizes of plastic balls, 55-gallon barrels, and fire hose rope/swings. Chimpanzees were also provided with daily foraging opportunities, enrichment devices, positive human interaction, and positive reinforcement training [12,13,14,15,35].

### 2.2. Procedure

Behavioral observations served as the basis for the procedures of the four studies. One observer (SNW) familiar with each chimpanzee and trained in chimpanzee behavioral data collection, collected live observations using 15-min focal animal sampling (Altmann, 1974) on a laptop computer running Noldus Observer XT 10 (Noldus, Wageningen, The Netherlands,) between 0700 and 1600 from July 2016 to May 2018. An ethogram of behaviors was constructed from an existing chimpanzee ethogram with added definitions and behaviors taken from [36] and the Chimpanzee Care Manual [37]. This ethogram was used for each of the studies. Locomotion was operationally defined as time spent hanging, walking, brachiating, and/or climbing (see Table 1). Locomotion was only recorded if it was independent of a social context. For example, brachiating that occurred during a play bout was recorded as play, not locomotion, and walking that occurred during a foraging bout (i.e., manipulating the food item and bringing food to the mouth) was recorded as foraging, not locomotion. Overall, each chimpanzee was observed for a minimum of 22 observation sessions, although some studies included more observations per chimpanzee. Across the studies, a total of 3936 observations, or 984 h of behavioral data, were collected. The research conducted in this study complied with the approved protocols of the UTMDACC Institutional Animal Care and Use Committee and complied with the legal requirements of the United States and the American Society of Primatologists’ Principles for the Ethical Treatment of Primates.

### 2.3. Study-Specific Methods

#### 2.3.1. Locomotion and Changes in Space per Animal and Type of Housing (Previously Published)

We examined changes in behavior as a function of increases or decreases in living space and changes in the type of housing. For full methodological details, please see [13]. Briefly, 22 captive chimpanzees in three social groups were moved through three living space and enclosure scenarios. One group that was housed in a single Primadome™ (approximately 1000 ft2 total or 142 square feet per animal) was moved to a double Primadome™ (approximately 2000 ft2; 284 square feet per animal). A second group was moved from a single Primadome™ to a corral (approximately 4500 ft2 or 645 square feet per animal), and a third group was moved from a corral to a double Primadome™. Behavioral data were collected as described above for four months prior to the transfer (pre-transfer period) and five months following the transfer (post-transfer period). We used bootstrapped paired samples t-tests to examine within-subject changes in behavior pre- and post-transfer [13].

#### 2.3.2. Locomotion and Group Size and Group Composition (Previously Published)

We examined changes in behavior as a function of differences in group size and group composition. The group size was dichotomized along previous NIH group size guidelines [38] as either seven or more compared to six or fewer animals per group. Group composition included three categories: (1) average age of the group, defined as the mean age of all animals in the social group; (2) within-group age range, defined as the oldest animals’ age minus the youngest animal’s age within the group; and (3) percentage of males within the group, dichotomized as less than half males within the group compared to at least half males. Please see [15] for full methodological details. Focal animal observations on all 120 chimpanzees were conducted as described in the General methods above. A bootstrapped MANCOVA with group average age as a covariate was used to examine differences in behavior as a function of these social environment characteristics.

#### 2.3.3. Locomotion and Geriatric Group Status (Previously Published)

We compared the behavior of elderly (*n* = 35) chimpanzees housed in geriatric (average age of the group ≥35 years) and non-geriatric (≤34 years) social groups. Compared to non-geriatric groups, geriatric groups were characterized by smaller size, smaller within-group age ranges, fewer males within the group, a higher mean age of the group, and a larger number of chimpanzees that exhibited impairments in mobility. Please see [12] for full methodological details. Focal animal observations on the 35 chimpanzees were conducted as described in the General methods above. A multivariate ANCOVA with impairment mobility scores as a covariate was used to examine behavioral differences between elderly chimpanzees housed in geriatric groups and those housed in non-geriatric groups.

#### 2.3.4. Locomotion and Voluntary Participation in a Medication Choice Paradigm (Previously Published)

As part of a study examining chimpanzee preferences for medications to treat arthritis, we examined changes in behavior as a function of voluntary participation in the medication choice protocol. For full methodological details, please see [14]. Briefly, four arthritic chimpanzees that were receiving daily analgesic medication to treat the symptoms of their arthritis and their mobility impairments were chosen as participants for a medication choice paradigm. In the paradigm, chimpanzees were assigned to one of two groups that determined the order of medications (either ibuprofen or meloxicam) that they would receive in either blue or green Gatorade. The first phase was a baseline phase in which the chimpanzees received their normal analgesic medication (meloxicam in pill form). In the second, third, fourth, and fifth phases (following an ABBA design), two chimpanzees received ibuprofen in blue Gatorade (A), then meloxicam in green Gatorade (BB), then ibuprofen again in blue (A), respectively. The remaining two chimpanzees received ibuprofen in green and meloxicam in blue Gatorade. This procedure allowed the chimpanzee to form an association between the color of the Gatorade and the way that that color made them feel (based on which medication was included in that color). In the final phase of the study (the choice phase), each chimpanzee was allowed to choose the color of the Gatorade (and thus, which medication) they preferred each morning for one month. During each of these phases, behavioral data were collected as described above in the General Method section. Given the small sample size, we used nonparametric Friedman’s rank tests with Wilcoxon posthoc tests for within-group comparisons across phases [14].

#### 2.3.5. Correlational Data between Locomotion and Other Welfare-Based Behaviors (Unpublished Data)

We used Pearson’s bivariate correlation to explore the relationships between locomotion and other behaviors that are often used as indicators of welfare, including social play, social grooming, self-grooming, scratching, abnormal behavior, and inactivity (see Neal Webb et al., 2019 for full ethogram). Briefly, inactivity included “inactive rest” (i.e., immobile, generally relaxed) and “inactive alert” (awake and alert but not involved in any active behavior such as locomotion, foraging, etc.). Abnormal behavior was a composite measure that included regurgitation and reingestion, feces smearing, idiosyncratic body movement (e.g., rocking), idiosyncratic body manipulation (e.g., thumb-sucking), hair plucking, and any other abnormal behavior not listed (see [13] for supplementary ethogram). We also examined the relationship between each chimpanzee’s behavioral diversity score (the average number of different positive welfare-related behaviors exhibited by the subjects during an observation period) and their level of locomotion using Pearson’s bivariate correlation. The calculations for behavioral diversity are described in detail in [38]. Briefly, to calculate this score, the number of different, species-typical behaviors exhibited by the chimpanzee was counted, excluding negative welfare-related behaviors (i.e., abnormal behaviors, rough-scratching), which ensured that behavioral diversity would not be confounded by the possibility that the increase in diversity was due to the addition of an abnormal behavior to the repertoire. Therefore, increases in this measure signified an increase in the number of species-typical behaviors that comprised the observed repertoire of the subject [39].

### 2.4. Data Analyses

First, the total duration of “out-of-view” for each observation for each chimpanzee was subtracted from the total observation time (900 s) to create total “within-view” durations (chimpanzees were in-view an average of 98% of each observation). Then, the total durations of each behavior for each chimpanzee were summed across all observations for that chimpanzee and divided by the total in-view duration time to create average durations of behavior. These averages were then converted into percentages ((average behavior duration in seconds/in-view duration in seconds) ∗ 100). All analyses were performed in SPSS Statistics 22 (IBM Corporation, Chicago, IL, USA). The significance level for all analyses was set at *p* < 0.05.

## 3. Results

### 3.1. Locomotion and Changes in Space per Animal and Type of Housing (Previously Published)

**Figure 1:**
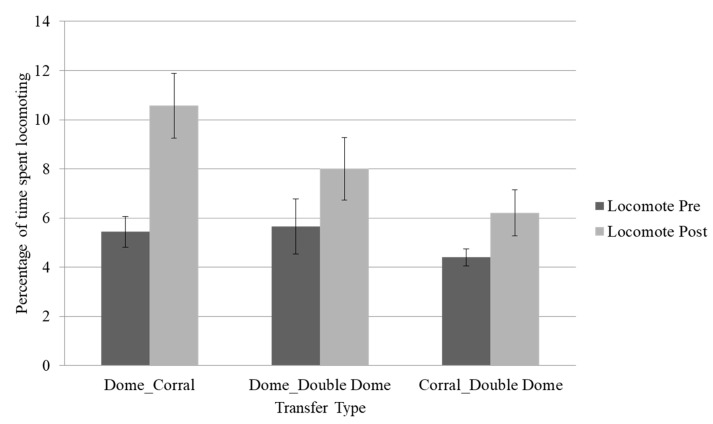
Mean percentage of time spent locomoting across the three transfer types. Error bars represent the standard error of the mean. Locomotion was trending higher post-transfer in the dome (~142 square feet per animal) to corral (~645 square feet per animal, *p* ≤ 0.08) and significantly higher in the dome to double dome (~284 square feet per animal, *p* ≤ 0.05) transfers, but not significantly so in the corral to double dome transfer (*p* > 0.10).

Locomotion was higher post-transfer across all transfer types, regardless of increases or decreases in space per animal or the type of change in housing (although not significantly so in the group that transferred from the corral to the double dome). As shown in Figure 1, chimpanzees exhibited up to a 5% increase in locomotion following transfer. Furthermore, these increases in locomotion were accompanied by significant decreases in rough scratching in the group that moved from the dome to the corral (*p* < 0.05) and by increases in behavioral diversity scores across all transfer types (*p* < 0.05) [13].

### 3.2. Locomotion and Group Size and Group Composition (Previously Published)

**Figure 2:**
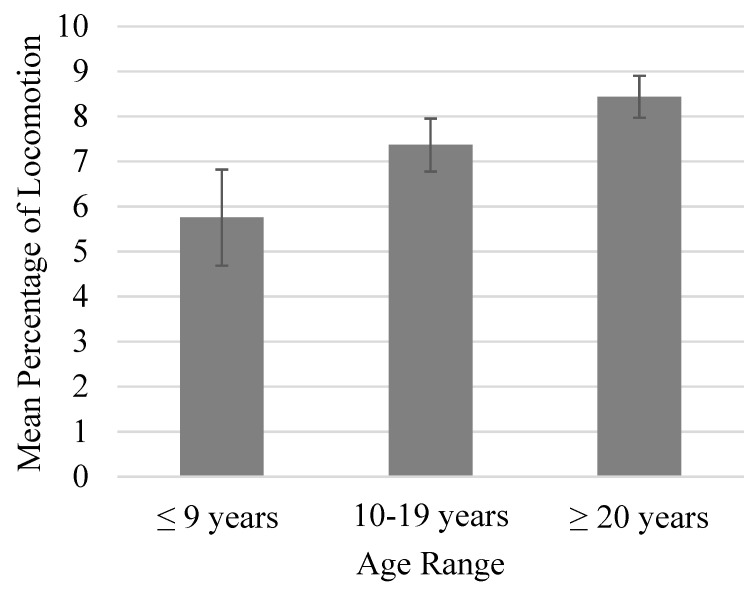
Mean percentage of time spent locomoting as a function of within-group age range. Error bars represent the standard error of the mean. Locomotion was trending higher in groups with a larger within-group age range (*p* = 0.067).

**Figure 3:**
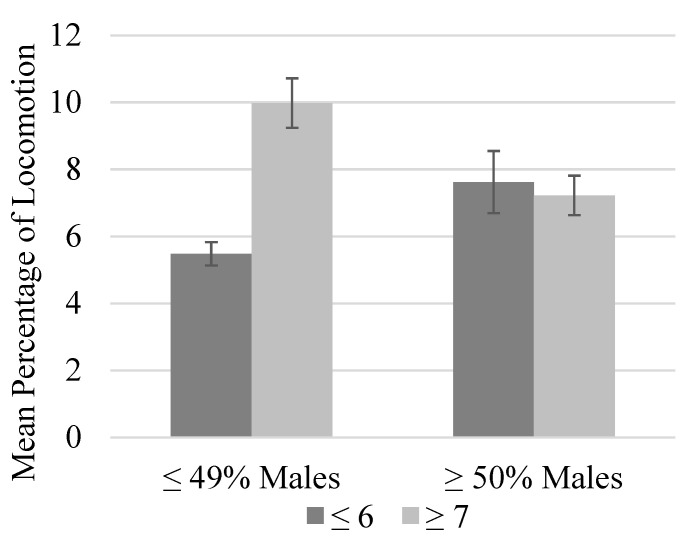
Mean percentage of time spent locomoting as a function of group size and percentage of males in the group. Error bars represent the standard error of the mean. Within smaller groups (six or fewer chimpanzees), locomotion was significantly higher when at least half the chimpanzees in the group were males (*p* ≤ 0.05). Within larger groups (seven or more chimpanzees), the mean percent time spent locomoting was significantly higher in groups that comprised less than half male chimpanzees (*p* ≤ 0.05). Lastly, the mean time spent locomoting was highest in larger groups that contained less than half male chimpanzees (*p* ≤ 0.05).

There was a trend toward higher levels of locomotion in groups with a larger within-group age range (*p* = 0.067; Figure 2). Furthermore, these changes coincided with lower levels of inactivity (*p* < 0.05). Locomotion was similar across group sizes if the group contained at least 50% males, but locomotion was approximately 4.5% higher in larger groups (≥7) that contained less than 50% males (Figure 3) [15].

### 3.3. Locomotion and: Geriatric Group Status (Previously Published)

**Figure 4:**
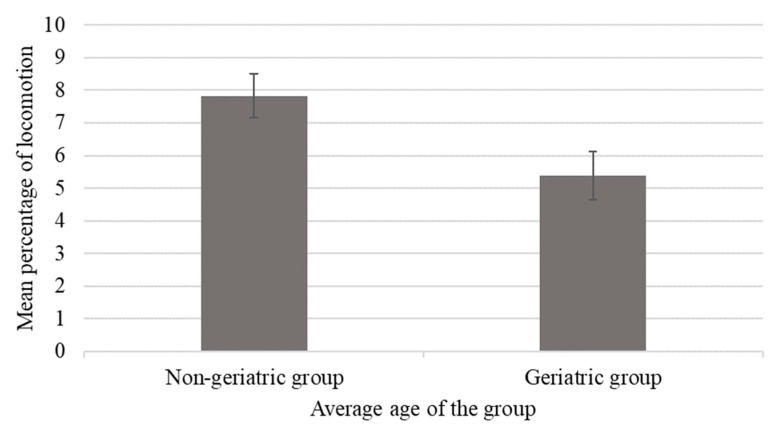
Mean percentage of time spent locomoting as a function of housing in a geriatric (group average age greater than or equal to 35 years) compared to non-geriatric (group average ages less than 35 years) groups. Error bars represent the standard error of the mean. Elderly chimpanzees housed in non-geriatric groups spent more time locomoting than those housed in geriatric groups (*p* ≤ 0.05).

Among elderly chimpanzees, those housed in geriatric groups (average age of group members >35 years) showed lower percentages of locomotion compared to those housed in non-geriatric groups (average age of group members ≤35 years; *p* < 0.05; Figure 4) [12], and this difference in behavior coincided with lower levels of rough scratching and submissive behaviors (*p* < 0.05).

### 3.4. Locomotion and Voluntary Participation in Medication Choice (Previously Published)

**Figure 5:**
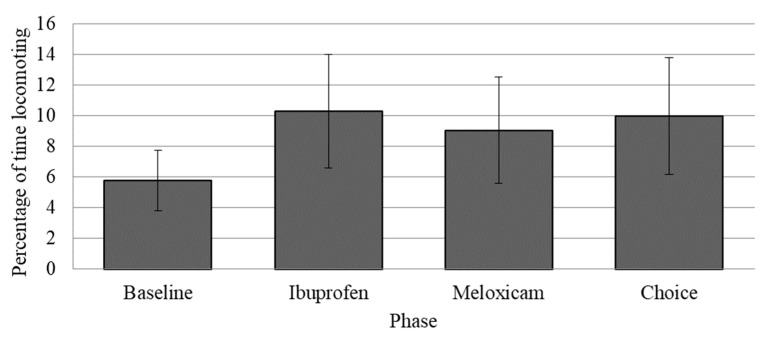
Mean percentage of time spent locomoting as a function of the experimental phase in the medication choice study. Error bars represent the standard error of the mean. The percentage of time spent locomoting was higher during the experimental phases of the paradigm, including the choice phase (during which chimpanzees received a choice between meloxicam and ibuprofen) compared to the baseline phase (prior to the start of the study) (*p* ≤ 0.05).

Locomotion was significantly higher across all study phases (when subjects received their arthritis medication dissolved in Gatorade) compared to the baseline phase (when subjects received meloxicam in pill form, see Figure 5). There were also decreases in rough scratching during the choice phase compared to the other phases (*p* < 0.05) [14].

### 3.5. Correlational Data between Locomotion and Other Welfare-Based Behaviors (Unpublished Data)

**Figure 6:**
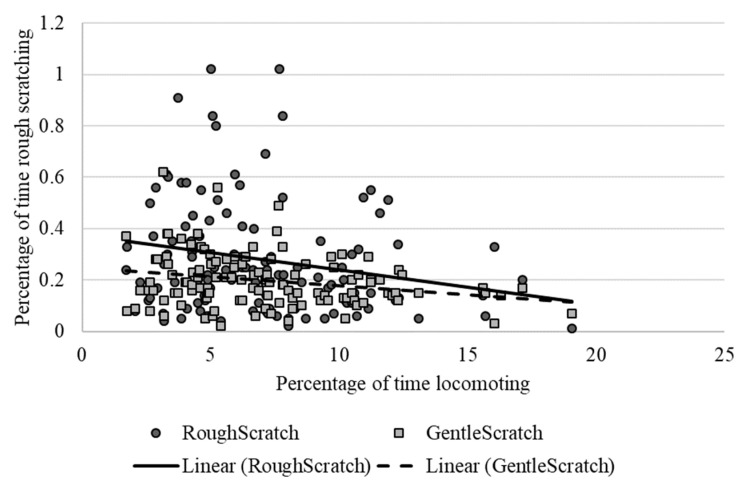
Negative relationship between the mean percentage of time spent locomoting and the percentage of time spent rough scratching (dark circles and solid trend line) and gentle scratching (light squares and dotted trendline). Chimpanzees that exhibited higher levels of scratching showed lower levels of locomotion.

**Figure 7:**
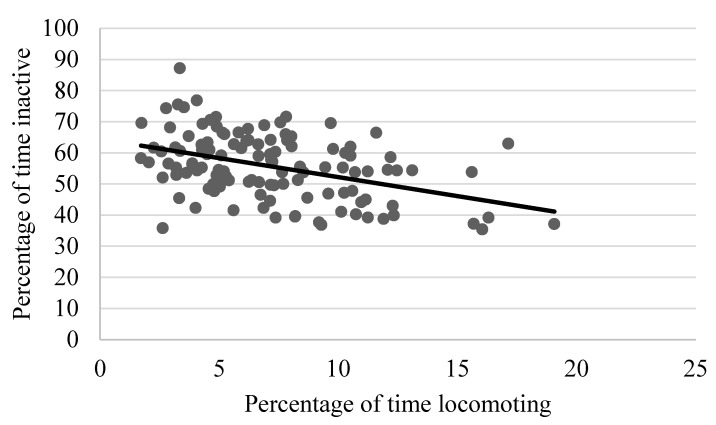
Negative relationship between the mean percentage of time spent locomoting and time spent inactive. Chimpanzees that spent more time inactive exhibited lower levels of locomotion (*p* ≤ 0.05).

**Figure 8:**
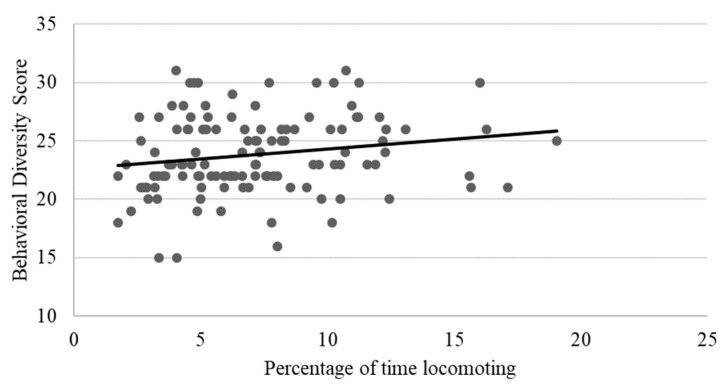
Positive relationship between behavioral diversity scores and mean percentage of time spent locomoting. Chimpanzees that spent more time locomoting had higher behavioral diversity scores (i.e., exhibited a higher number of different positive welfare behaviors) (*p* ≤ 0.05).

**Figure 9:**
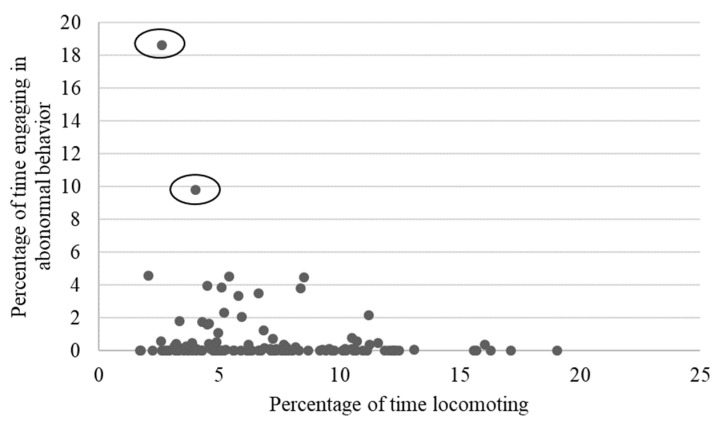
Relationship between mean time spent engaging in abnormal behaviors and mean percentage of time spent locomoting. Once the two outliers (circled) were removed, the relationship was no longer significant (*p* = 0.10).

As shown in Table 2, locomotion showed weak to moderate, yet still statistically significant correlations with several behaviors, including negative relationships with rough scratching, gentle scratching (Figure 6), and inactivity (Figure 7), and a positive correlation with behavioral diversity scores (Figure 8; *p* ≤ 0.05). There was also a statistically significant negative relationship between abnormal behavior and locomotion (*p* = 0.03; Figure 9). Upon further inspection of the data, two outliers seemed to be skewing the relationship (one chimpanzee that displayed rocking behavior and another that displayed thumb-sucking). Upon removal of these two outliers, the relationship between abnormal behavior and locomotion was no longer significant (*p* = 0.10).

## 4. Discussion

This paper summarizes and extends findings related to chimpanzee locomotion in captivity, specifically in terms of (1) manipulations to certain physical and social housing conditions and (2) participation in experimental paradigms. Across these four studies, a pattern emerged that seems to show a meaningful relationship between locomotion and conditions related to enhanced welfare, including increased space per animal, changes in housing type, housing in more diverse social groups, and voluntary participation in a self-medication program. Furthermore, the increased locomotion observed in these studies in response to these positive environmental conditions coincided with changes in other “positive” welfare-related behaviors, including decreased inactivity levels and behavioral diversity, and decreased rough scratching and submissive behavior.

In two of the studies summarized here, we used changes in time spent in locomotion across experimental conditions to assess changes in well-being. For example, the increased time spent in locomotion following changes in space per animal and type of housing suggested an increase in well-being post-transfer. Similarly, the increased locomotion in Phases 2–6 of the medication choice study indicated an improvement in welfare from the baseline condition. In the other two studies, individual chimpanzees served in only one condition/group and well-being was compared across groups. The higher level of locomotion in groups with larger within-group age ranges, in larger groups with less than half males, and among elderly chimpanzees housed in non-geriatric groups suggested higher welfare of those groups compared to the other groups included in each respective study. These are similar, but also slightly different, comparisons, and locomotion was sensitive enough to differ in both types of assessments.

We also found a statistically significant positive correlation between locomotion and another behavioral indicator of positive well-being (behavioral diversity), a negative correlation with an indicator of stress (scratching), and a negative correlation with inactive behavior (a behavior typically associated with decreased welfare when levels are high). We also found a negative correlation between locomotion and abnormal behavior when including all individuals in the analysis. However, after removing two outliers from the analysis, this relationship was no longer significant. Given these outliers, and the fact that the level of abnormal behavior in our sample was very low (mean = 0.67, SEM = 0.19, median = 0.00), this particular result should be interpreted with caution (although it is interesting to note that the two outliers that were removed from the analysis showed low levels of locomotion, one in the bottom 10th percentile and the other in the 20th). We did not find relationships with social play or grooming behaviors, which are often associated with positive well-being. Regardless, these relationships with other behavioral welfare indicators provide some additional evidence supporting the use of locomotion as an indicator of positive well-being.

There are several benefits to the use of locomotion as a welfare indicator. First, there are fewer widely accepted behavioral indicators of positive welfare in chimpanzees (e.g., social play) than there are of negative welfare (abnormal behavior, self-directed behavior, rough scratching), and there has been a push toward the validation and increased use of positive welfare indicators [40,41]. Based on the current findings, along with some of the research summarized earlier, we suggest that locomotion can be profitably added to the small list of positive welfare-related behavioral indicators available for use in behavioral research studies. Second, locomotion seems to be more sensitive to environmental differences or experimental changes than other measures of well-being. For example, rough scratching, abnormal behavior, and social play often occur at such low rates within individuals (sometimes below 1%) that statistical analyses can be difficult to perform, and/or the differences in levels across conditions are so small that they are not necessarily meaningful [10,36,42,43,44]. On the other hand, baseline levels of locomotion in captive chimpanzees range from 5 to 12% [8,9,10,11,16]. Changes across conditions and/or differences across groups in time spent in locomotion in the studies reported here were between 2 and 5%, equating to a difference of between 14 and 36 min per day of additional walking, climbing, hanging, and/or brachiating. In an aging chimpanzee population that is experiencing health-related issues (e.g., obesity, arthritis, cardiovascular disease, etc.), an average of an extra 20 min per day of voluntary movement can be an impactful and meaningful enhancement to welfare.

Although locomotion occurs at higher rates than some other behavioral welfare indicators, levels of locomotion would likely increase only to a certain point under captive conditions. Time spent in locomotion in captivity is, under current management practices, going to be lower than time spent in locomotion for wild chimpanzees [8,9,10,11]. Wild chimpanzees must travel to search for and access required resources (e.g., food, sleeping sites, water, patrols, etc.) [3]. On the other hand, captive chimpanzees are provided the majority of these resources [25]. It is possible that, when NHPs (including chimpanzees) do not need to move to access resources, they simply do not. As such, we should not expect locomotion to increase to a level that is comparable to that of wild chimpanzees following attempted enhancements to welfare.

It is important to note that the relationship between locomotion and welfare is nuanced. First, the meaning or implication of changes in locomotion is context-dependent. For example, increases in locomotion in response to increased space per animal may be considered indicative of positive welfare, whereas increased locomotion in response to a predator is indicative of tension or anxiety [31,32,45]. As such, it is important to consider the context when making conclusions about the meaning of the overall level of, or change in, locomotion in response to a manipulation. Second, locomotion as a welfare indicator must be distinguished from other behaviors that may resemble the behaviors that comprise locomotion. For example, stereotypic or repetitive locomotion (i.e., pacing) is indicative of poor welfare [6,46]. As such, locomotion must be precisely operationally defined so as not to include behaviors such as pacing or agitated locomotion (e.g., [6,46]). In the current studies, the operational definitions in the ethogram included only normal locomotive behaviors, including walking, climbing, brachiating, and hanging, which were not characterized by the repetitive, unfocussed movement that distinguishes pacing behavior. In fact, pacing was purposefully excluded from locomotive behaviors, as it was included under “idiosyncratic movement” as an abnormal behavior [13]. Additionally, locomotion was further demarcated to exclude “agitated locomotion”, which was defined as “brisk, or rapid walking that often occurs with increased vigilance toward event, animal, or object” [13]. Therefore, these findings reflect normal locomotion and do not reflect pacing or other forms of locomotion that are typically related to negative welfare states. Lastly, it is worth noting that locomotion has been found to be unrelated to certain measures of *physical* well-being. Previous studies have found that rates of locomotory behavior were not significantly correlated with scores on the Mobility Scoring System (MSS) [12,14] or Fluency Scoring System [47], both of which reflect ease of movement. As such, it may be that, although chimpanzees’ ease of movement may decrease (for example, with age, injury, or illness), their rates of locomotory behavior remain stable [47].

## 5. Conclusions

We would like to note that we are not specifically advocating for particular changes to characteristics of the physical or social environment, or certain health or behavioral study practices (although the data across the studies outlined here do suggest some potential for welfare enhancements in these areas). Our purpose was to summarize and link findings regarding locomotion across the several published studies from our facility to highlight the pattern that emerged. We suggest that locomotion while considering contextual factors, may be a sensitive, and perhaps underutilized, measure of well-being in studies examining captive chimpanzee welfare. Overall, locomotion is easily observable, is of appreciable duration (allowing for robust statistical analyses), and is typically relatively easy to record, all of which make it a useful measure. As such, we are advocating for empirical evaluations that include time spent in locomotion as an indicator of well-being. Furthermore, since it is likely that previous behavioral studies included measures of normal locomotion in their ethograms (as these studies did), retrospective analyses of locomotion from such studies would also be useful.

## Figures and Tables

**Table 1:** Ethogram of locomotive behaviors.

Ethogram of Locomotive Behaviors	
Hang	All of the animal’s weight is supported by wire of walls (i.e., animal is grasping wire with hands and feet), or the animal is hanging beneath the climbing bars.
Walk	Moving through space at a calm, steady pace on horizontal surface (may be on ground, plank, or platform). If walking occurs with play face, then it is considered play. Includes walking with food. Includes bipedal walking as a means of travel from one point to another.
Climb	Individual is ascending from one point to another in normal location (e.g., onto a platform, from top of cage, to side of cage, etc.). If this occurs with play face, then it is considered play. Includes climbing with food.
Brachiate	Animal uses arms to swing from one location to another. If this occurs with play face, then it is considered play. Includes brachiating with food.

**Table 2:** Relationships between locomotion and welfare-related behaviors.

Behavior	r	*p*-Value
Social Play	0.01	0.88
Social Groom	−0.10	0.30
Groom self	−0.05	0.60
Rough Scratch **	−0.21	0.02
Gentle Scratch **	−0.25	0.01
Abnormal Behavior **	−0.20	0.03
Abnormal Behavior (excluding 2 outliers, *n* = 118)	−0.15	0.10
Inactive **	−0.42	0.00
Behavioral Diversity Score **	0.18	0.05

Note: *n* = 120 for all analyses unless otherwise noted. r = Pearson’s correlation coefficient. ** Statistically significant.

## Data Availability

Data used in the four published studies as well as the correlational data included in this manuscript are available upon reasonable request. Please contact the corresponding author.
